# Hemoclot Thrombin Inhibitor Assay and Expected Peak-Trough Levels of Dabigatran: A Multicenter Study

**DOI:** 10.3389/fcvm.2022.894888

**Published:** 2022-07-22

**Authors:** Zhiyan Liu, Guangyan Mu, Qiufen Xie, Hanxu Zhang, Jie Jiang, Qian Xiang, Yimin Cui

**Affiliations:** ^1^Department of Pharmacy, Peking University First Hospital, Beijing, China; ^2^School of Pharmaceutical Sciences, Peking University Health Science Center, Beijing, China; ^3^Department of Cardiology, Peking University First Hospital, Beijing, China; ^4^Institute of Clinical Pharmacology, Peking University, Beijing, China

**Keywords:** dabigatran, hemoclot thrombin inhibitor, quantitative detection, drug safety, cardiovascular medicine

## Abstract

**Purpose:**

Dabigatran concentrations monitoring are gaining importance of special situations, but limited data are available for the expected peak and trough levels. The hemoclot thrombin inhibitor (HTI) is dabigatran-calibrated quantitative determination of dabigatran concentration. This study aims to validate HTI assay as the quantification choice of dabigatran, and providing the expected peak and trough levels.

**Materials and Methods:**

This is a multi-center methodology validate study, including seven hospitals from Beijing, Shanghai, Henan, Hunan, Chongqing, and Fujian. We retrospectively analyzed plasma samples taken from 118 healthy subjects and 183 patients receiving dabigatran. Dabigatran concentrations were measured with HTI assay and high-performance liquid chromatography-tandem mass spectrometry (HPLC-MS/MS). Linear regression, Spearman correlation and Bland-Altman analysis were used in this study.

**Results:**

The mean concentration ratio of HPLC-MS/MS and HTI assays was 1.03 and 0.98 at 2 and 12 h, and the acceptance ranges for both the ratio limit as well as the limit of agreement were met, suggesting good agreement between the HTI-derived plasma concentrations and HPLC-MS/MS. The reference detection range of single dose dabigatran 150 mg in healthy subjects was 33–159 ng/ml. About 500 blood samples were taken from 183 patients suggested that the expected peak and trough levels range of dabigatran 110 mg was about 95–196 and 36–92 ng/ml.

**Conclusion:**

Hemoclot thrombin inhibitor assay can be a good quantitative detection method of dabigatran. Expected peak and trough levels provide a basis for the rational use of dabigatran, and provide important Asian population data for the update of the international clinical guidelines for hematological testing.

**Clinical Trial Registration:**

[https://clinicaltrials.gov], identifier [NCT03161496].

## Highlights

– Hemoclot thrombin inhibitor (HTI) assay can be a good quantitative detection method of dabigatran in Asian population.
– The expected peak and trough levels (ng/mL) range derived from HTI assay of dabigatran 110 mg were 95–196 and 36–92.
– A multi-center quantitative assay of dabigatran was conducted for the first time in the Asian population.

## Introduction

Dabigatran etexilate (Pradaxa^®^) is a prodrug of dabigatran, a direct inhibitor of thrombin. It is approved for ischemic stroke and systemic embolism in patients with non-valvular atrial fibrillation (NVAF) prevention, and prevention/treatment of venous thrombosis and thromboembolism (1–3). Studies indicated that all oral anticoagulants seem to have similar pharmacokinetic variability and a narrow therapeutic range (4). Thus, oral anticoagulants, such as dabigatran, will require monitoring of anticoagulation to determine the dose that best meets individualized therapy. Anticoagulation reduces stroke risk in atrial fibrillation, yet a retrospective cohort study found that in patients with incident atrial fibrillation, race/ethnicity was independently associated with initiating any anticoagulant therapy and direct-acting oral anticoagulant use among anticoagulant initiators (5). Compared with White patients, the adjusted odds ratio (OR) of initiating any anticoagulant therapy was significantly lower for Asian [OR, 0.82; 95% confidence interval (CI), 0.72–0.94] and Black (OR, 0.90; 95% *CI* 0.85–0.95) patients. Moreover, more adverse events have emerged in recent years among patients of taking dabigatran, including gastrointestinal discomfort, bleeding, etc. (6). Thus, thrombolysis in ischemic stroke, major bleeding events or prior to urgent invasive treatments, a possibility of monitoring dabigatran is deemed to optimize its dosage regimens, so as to perioperative care in time and change urgently demanded thrombolytic therapy (7). Our team found that 30 new potential SNPs of 13 reported candidate genes (ABCB1, ABCC2, ABCG2, CYP2B6, CYP1A2, CYP2C19, CYP3A5, CES1, SLCO1B1, SLC22A1, UGT1A1, UGT1A9, and UGT2B7) were associated with dabigatran metabolism. Testing of coagulation in patients treated with non-vitamin K antagonist oral anticoagulants was described in non-Chinese populations (8, 9), but limited data are available for Chinese population. Therefore, more data of dabigatran detection in Chinese or Asian population are needed to guide clinical use of personal therapy of dabigatran.

Activated partial thromboplastin (APTT) or prothrombin time (PT) were low sensitivity, reagent-specific or coagulometer-specific variations render standard coagulation assays (10, 11). So, they are less suitable for dabigatran detection and just can be used for qualitative determination (12, 13). Drug concentration is usually determined by high-performance liquid chromatography coupled with tandem mass spectrometry (HPLC-MS/MS) method. However, only a small number of specialized laboratories carried out HPLC-MS/MS project of dabigatran (14, 15). The present studies demonstrate that dabigatran can be determined under real-world conditions using clot-based ecarin clotting time assay (ECT), hemoclot thrombin inhibitor (HTI), and chromogenic assays from patients on treatment (16–18). However, ECT is limited for the low availability and insufficient standardization (19, 20). The HTI is specifically calibrated for dabigatran, so as to be a quantitative determination of dabigatran concentration. In the guidelines of 2019 “International Council for Standardization in Haematology Recommendations for Hemostasis Critical Values, Tests, and Reporting (ICSH),” the plasma concentration ranges data of dabigatran are mainly from a few kinds of literature. Moreover, limited information exists on their performance and expected peak/trough levels, especially in their ability to accurately measure concentrations in the Chinese population.

Thus, we aimed to validate HTI assay as a quantification choice of dabigatran in the Chinese population and provided the expected peak and trough levels. The study analyzed consistency of plasma dabigatran levels between HIT assay and HPLC-MS/MS in healthy subjects, and expected peak-trough levels were recommended.

## Materials and Methods

### Study Protocol and Population

This study was based on a bioequivalence (BE) trial conducted in China, which was mainly performed to assess the BE of domestic generic dabigatran etexilate capsules, with reference to the original product (brand name: Pradaxa^®^) in healthy subjects. Based on the BE trial, we added PD parameter tests that were conducted during any one of the two reference periods. We investigated the consistency of HIT assay with HPLC in the Chinese healthy population, and also tested it in a patient population to provide a possible reference range. Seven hospitals from Beijing, Shanghai, Henan, Hunan, Chongqing, and Fujian participated in this study. The data presented in this study were from Pradaxa. The study protocol was approved by the ethics committee and the Institutional Review Board of Peking University First Hospital and all sub-central hospitals. Registration number in Clinical Trial system is NCT03161496.

Healthy volunteers were required to be 18–45 years old, with a Body Mass Index ranging from 18–26 kg/m^2^. Health status was confirmed *via* medical history interview, physical examination, vital signs and laboratory examination. Patients meeting the following criteria were included: (1) patients with NVAF using dabigatran for prevention of ischemic stroke and systemic embolism; (2) Age older than 18; (3) Baseline data were available.

### Pharmacokinetics Assessment

The venous blood samples were collected 0 h and post-dose at 16–19 time points in 3.0 ml EDTA-K2 tubes and centrifuged at 4°C for 10 min at 3,000 g. The plasma samples were stored at –80°C until analysis.

The total anti-thrombin effect from dabigatran etexilate is a result of dabigatran (alone) and dabigatran acylglucuronides. An assay for total dabigatran (the sum of free dabigatran and the contribution from dabigatran acylglucuronides) in plasma is developed, validated and applied (21, 22). Thus, dabigatran concentrations were determined by validated HPLC-MS/MS assays at Shimadzu 30 series, API 6500 (Applied Biosystems, Inc., American). The standard calibration curves with good linearity were built for dabigatran within the concentration range of 1.0–300.0 ng/mL, and the lower limit of quantitation (LOQ) was 1.0 ng/mL. The precision (% CV), intra-batch accuracy and inter-batch accuracy were within 6.2–9.2, 90.3–104.3, and 94.6–103.8%, respectively. PK parameters were analyzed by non-compartmental methods using Phoenix Win-Non-lin 7.0 (A Certara™ Company, Princeton, NJ, United States). C_max_ (Maximum concentration) and T_max_ (the time to C_max_) were obtained from the observed data, while terminal half-life (t_1/2_), plasma concentration vs. time from 0 to the last measurable time point (AUC_0–t_) were calculated.

### Pharmacodynamics Assessment

For detecting pharmacodynamics (PD) parameters, the sampling times were 0, 2, 8, 12 h after dabigatran administration respectively. In patients’ population, plasma sample obtained longer than 10 h after the previous dose was considered for trough concentration; average 2.0 h (range: 1.0–3.0 h) post dose was considered for peak concentration. The reference detection range refers to plasma dabigatran concentrations between the 2.5th and 97.5th percentiles of all values determined by HPLC-MS/MS.

Blood samples were collected *via* 2.7 ml sodium citrate tubes, then centrifuged for 15 min at 2,500 g at room-temperature. The plasma samples were stored at –70°C within 6 months until analysis.

Pharmacodynamic assessment was performed at a centralized facility in Peking University First Hospital. The HTI, PT, and APTT were measured on Sysmex CS-2100i (Sysmex, Kobe, Japan). PT and APTT were tested using Coagulation Method Assay Kits (Thromborel-S^®^ and Actin^®^, Marburg, Germany). The normal reference range for the PT and APTT assays used in this study are 10.1–12.6 s for PT and 26.9–37.6 s for APTT. Concentration detection is divided into two standard curves, one is suitable for normal and slightly high concentration range, another one is suitable for low concentration range. When the concentration is below 120 ng/ml, the low concentration standard curve is switched to obtain a more accurate concentration value. PK assessment was done in each sub-center, without significant matrix effect found in any sub-center. PD assessment was all performed in our hospital.

### Statistical Analyses

Statistical analyses were conducted through Statistical Package for Social Sciences (SPSS version 22.0, SPSS Inc., Chicago, IL, United States). Correlation of coagulation assays and HPLC-MS/MS was performed using linear regression, Spearman correlation analysis (*p* < 0.05 means significant correlation). Difference between HPLC-MS/MS and specific dabigatran assay results was calculated by Bland-Altman analysis. Mean ± standard deviation (SD), frequencies and percentages were presented for continuous and categorical variables respectively.

## Results

### Demographic Characteristics

Total 118 healthy subjects taking single dose dabigatran 150 mg were enrolled. Demographics characteristics are provided in Table 1. No significant difference was found in the baseline.

**TABLE 1:** The baseline characteristics, pharmacokinetics (PK) and pharmacodynamics (PD) parameters result of enrolled healthy subjects.

Dabigatran	Results
Dose, mg	150
N	118
Gender (Male, %)	72
Age, years	24.02 ± 5.55
BMI, kg/m^2^	22.21 ± 1.65
C–0 h, ng/mL	0.00 ± 0.00
C–2 h, ng/mL	85.51 ± 73.77
C–8 h, ng/mL	57.60 ± 23.46
C–12 h, ng/mL	36.40 ± 3.15
t_1/2_, h	8.78 ± 1.33
T_max_, h	3.61 ± 2.09
C_max_, ng/mL	139.99 ± 55.61
AUC_0–t_, ng × h/mL	1247.43 ± 442.08
AUC_0–12_,ng × h/mL	1285.24 ± 441.79
APTT–0 h, s	27.78 ± 3.50
APTT–2 h, s	39.69 ± 10.69
APTT–8 h, s	38.52 ± 6.00
APTT–12 h, s	35.22 ± 5.37
PT–0 h, s	11.74 ± 1.88
PT–2 h, s	12.83 ± 1.58
PT–8 h, s	12.44 ± 1.31
PT–12 h, s	11.97 ± 0.92
HTI–0 h, ng/mL	2.99 ± 2.37
HTI–2 h, ng/mL	88.89 ± 79.08
HTI–8 h, ng/mL	61.91 ± 27.46
HTI–12 h, ng/mL	40.00 ± 16.63

*BMI, body mass index; HTI, hemoclot thrombin inhibitor; APTT, activated partial thromboplastin; PT, prothrombin time; AUC, area under the curve. Reference ranges for PT 10.1–12.6 (s), and APTT 26.9–37.6 (s).*

About 205 patients taking dabigatran 110 mg bid were included in the study, but, 22 people were lost to follow-up or blood samples were not collected. Thus, 183 patients were finally included in our analysis. The average age was 72.11 ± 9.86 (mean ± SD) years old, and 59.6% was male. The baseline of fibrinogen, D-dimer, TT, creatinine, glomerular filtration rate, etc., were provided in Table 2.

**TABLE 2:** Baseline characteristics and pharmacodynamics (PD) parameters result of the patients with dabigatran.

Dabigatran dose	Target range	Results
N	–	183
Dose (mg)	–	110
Gender (Male,%)	–	59.6
Age, years	–	72.11 ± 9.86
BMI, kg/m^2^	–	26.74 ± 16.93
APTT-0 h, s	26.9–37.6	36.47 ± 8.40
APTT-peak, s	26.9–37.6	47.89 ± 4.43
APTT-trough, s	26.9–37.6	39.08 ± 9.74
PT-0 h, s	10.1–12.6	15.58 ± 5.90
PT-peak, s	10.1–12.6	14.04 ± 3.50
PT-trough, s	10.1–12.6	13.79 ± 6.15
HTI-0 h, ng/mL	–	4.06 ± 2.92
HTI-peak, ng/mL	–	145.57 ± 50.16
HTI-trough, ng/mL	–	63.74 ± 27.95
HGB, g/L	110–160	133.95 ± 17.55
PLT, 10^9^/L	100–300	206.55 ± 91.07
MPV, fL	7.50–11.5	11.61 ± 13.29
TG, mmol/L	0.56–1.70	1.32 ± 0.63
TCHO, mmol/L	3.40–5.20	5.98 ± 27.97
LDL, mmol/L	2.10–3.10	2.23 ± 0.81
HDL, mmol/L	1.00–1.55	1.12 ± 0.34
FIB, g/L	2.00–4.00	2.85 ± 0.73
D-dimer, μg/L	<200	530.81 ± 57.55
TT, s	12.5–16.5	47.00 ± 61.87
HbA1c, %	4.00–6.00	6.27 ± 0.99
T3, nmol/L	1.80–2.90	1.54 ± 0.36
T4, nmol/L	66.0–181	101.33 ± 25.47
CREA, umol/L	53.0–115	83.49 ± 19.34
GFR, mL/min	–	73.03 ± 17.19
ALT, IU/L	7.00–50.0	23.05 ± 15.16
AST, IU/L	13.0–40.0	24.76 ± 21.48
HAS-BLED	–	2.16 ± 1.04
CHA2DS2-VASc	–	3.79 ± 1.70

*BMI, body mass index; HTI, hemoclot thrombin inhibitor; APTT, activated partial thromboplastin; PT, prothrombin time; HGB, hemoglobin; PLT, platelet; MPV, mean platelet volume; TG, triglyceride; LDL, low density lipoprotein; HDL, high density lipoprotein; FIB, fibrinogen; TT, thrombin time; T3, triiodothyronine; T4, thyroid hormone; CREA, creatinine; GFR, glomerular filtration rate; ALT, alanine aminotransferase; AST, aspartate aminotransferase.*

### Correlation of Hemoclot Thrombin Inhibitor With Blood Concentration of HPLC-MS/MS

In healthy subjects, mean concentration (ng/mL) of 2, 8, and 12 h after taking dabigatran 150 mg (single dose) *via* HPLC-MS/MS were 85.51, 57.60, and 36.40. The mean t_1/2_, T_max_ and C_max_ were 8.78 h, 3.61 h, and 139.99 ng/ml. The HTI assay of mean dabigatran concentration (ng/ml) of 2, 8, and 12 h were 88.89, 61.91, and 40.00 (Table 1). Thus, in our study, the reference detection range of single dose dabigatran 150 mg in healthy subjects was 33–159 ng/ml.

**FIGURE 1:**
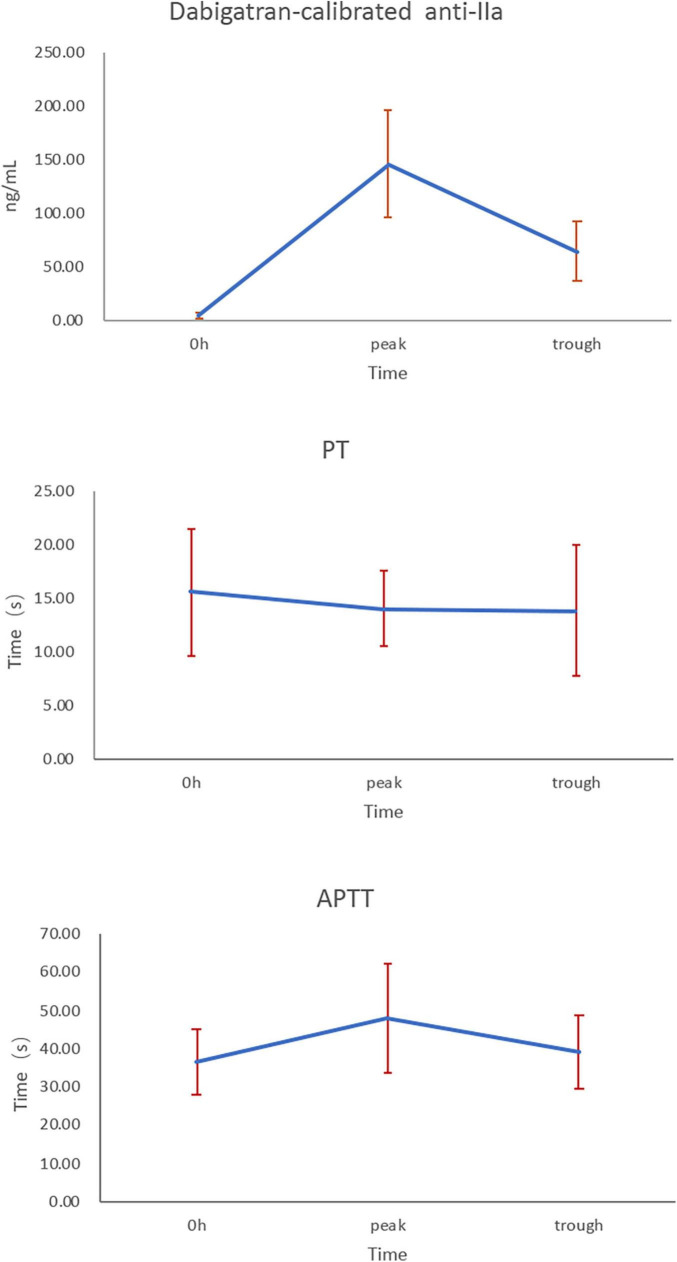
Bland-Altman difference plot analysis between HPLC-MS/MS and HTI at 2 and 12 h.

Bland-Altman difference plot analysis was used to assess agreement between the HIT test method and the reference method LC-MS/MS. The mean concentration ratio of LC-MS/MS and HTI assays was 1.03 and 0.98 at 2 and 12 h, thus fulfilling the suggested acceptance criterion for bioanalytical sample analysis (23); ratio limit range was 0.98–1.08, 0.86–1.12 for 2 and 12 h (acceptance range, 0.83–1.20); and limit of agreement was 0.89–1.18, 0.80–1.19 for 2 and 12 h (acceptance range, 0.83–1.20). The acceptance ranges for both the ratio limit as well as the limit of agreement were met, suggesting good agreement between the HTI-derived plasma concentrations and LC-MS/MS (Figure 1). In addition, correlation and linear regression analysis of PT, APTT, HTI with drug concentration measured by HPLC-MS/MS in healthy subjects were done in the study. APTT showed significant correlation with concentration of dabigatran at 2, 8 and 12 h (*p* = m 5.41 × 10^–28^, 0.90 × 10^–4^, 7.90 × 10^–5^); PT had correlation with concentration of dabigatran only at 2 h (*p* = 1.90 × 10^–5^); HTI showed significant correlation with concentration of dabigatran at 2, 8, and 12 h (*p* = 5.97 × 10^–55^, 3.97 × 10^–32^, 1.48 × 10^–27^). In the linear regression analysis, APTT consistent with concentration of dabigatran only at 2 h (*p* = 0.02); PT had no consistency with concentration of dabigatran (*p* > 0.05); HTI had significant consistency with concentration of dabigatran at 2, 8, and 12 h (*p* = 0.20 × 10^–3^, 7.64 × 10^–12^, 9.13 × 10^–17^, *r* = 0.94, 0.84, 0.80, Table 3). HIT had good correlation with HPLC-MS/MS at all collection time (*p* < 0.001), suggesting that it was good quantitative detection method of dabigatran in Asian population.

**TABLE 3:** Correlation and linear regression analysis of prothrombin time (PT), activated partial thromboplastin (APTT), hemoclot thrombin inhibitor (HTI) with drug concentration measured by HPLC-MS in healthy subjects.

Methods	Correlation analysis				Linear regression analysis			
		
Time		2 h	8 h	12 h		2 h	8 h	12 h
APTT	*r*	0.80**	0.40**	0.36**	Beta	0.24	–0.04	–0.02
	*p*	5.41E–28	0.90E–4	7.90E-5	*t*	2.40	–0.61	–0.22
					*p*	0.02*	0.55	0.82
PT	*r*	0.38**	0.21*	0.13	Beta	–0.08	0.12	0.03
	*p*	1.90E–5	0.03	0.16	*t*	–1.16	1.92	0.51
					*p*	0.25	0.06	0.61
HTI	*r*	0.94**	0.84**	0.80**	Beta	0.49	0.64	0.77
	*p*	5.97E–55	3.97E–32	1.48E–27	*t*	4.57	7.70	9.84
					*p*	0.20E–3**	7.64E–12**	9.13E–17**

HTI, hemoclot thrombin inhibitor; APTT, activated partial thromboplastin; PT, prothrombin time; **Correlation is significant at the 0.01 level (2-tailed); *Correlation is significant at the 0.05 level (2-tailed). r means correlation coefficient.

**FIGURE 2:**
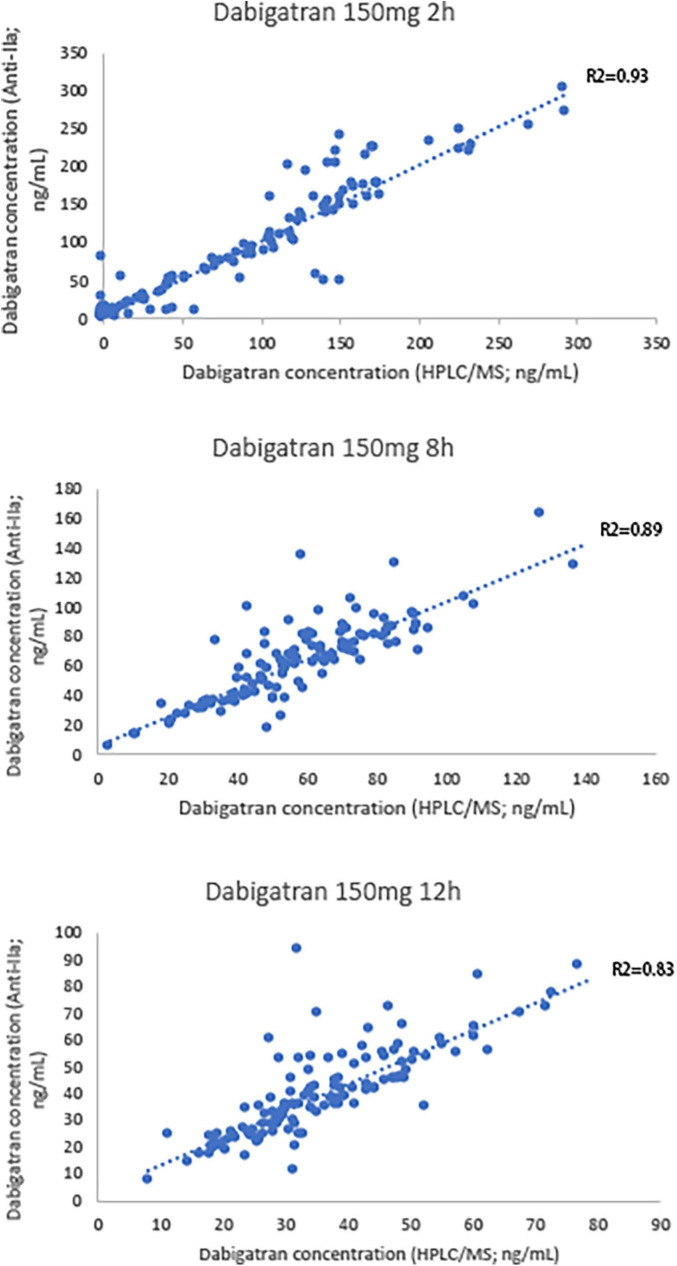
Scatter plot of hemoclot thrombin inhibitor (HTI) and blood concentration of HPLC-MS/MS.

Scatter plot of HTI and blood concentration of HPLC-MS/MS is showed in Figure 2. Difference between HPLC-MS/MS and HTI was calculated using Bland-Altman plot analysis at 2 and 12 h (Figure 2).

### Hemoclot Thrombin Inhibitor Assay in Patients on Dabigatran

**FIGURE 3:**
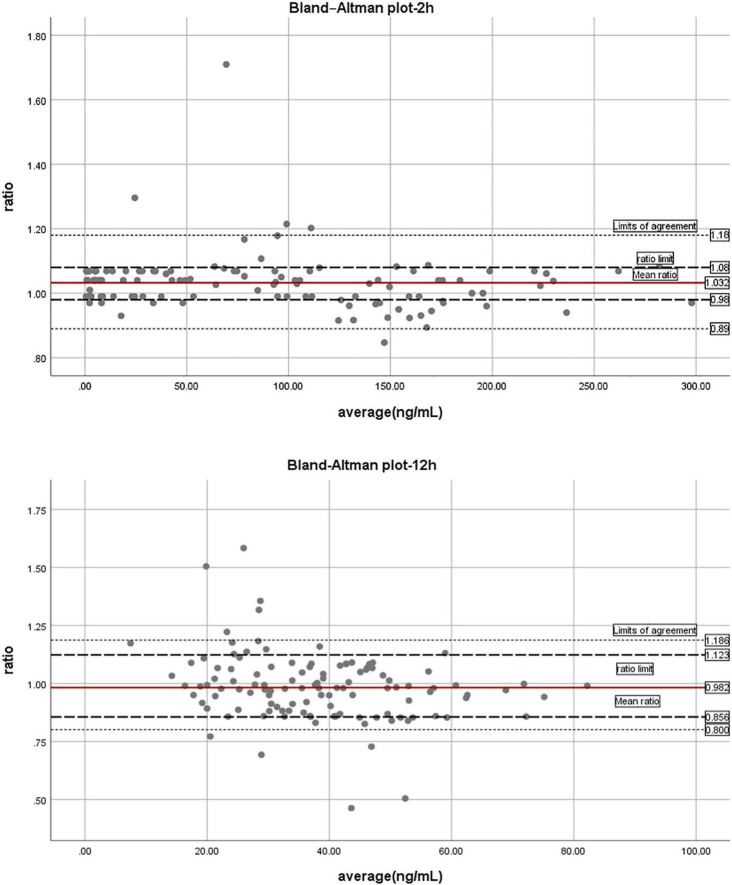
The trend chart of pharmacodynamic parameters at different blood collection points of dabigatran 110 mg.

After blood collection, HTI, APTT, and PT were detected in the enrolled 183 patients (Table 2). The mean concentration of dabigatran 110 mg bid at pre-dose, peak and trough were 4.06, 145.57, and 63.74. APTT (s) was 36.47, 47.89, and 39.08; PT (s) was 15.58, 14.04, and 13.79, respectively. PD parameters at different collection points of dabigatran 110 mg is shown in Figure 3. Compared with healthy subjects’ HTI results, patients taking lower dose had higher values, especially in peak concentration. The above indicated that dabigatran concentration should be monitored in specific clinical practice. HTI assay of about 500 blood samples from 183 patients suggested that the expected peak and trough levels range of dabigatran 110 mg bid was about 95–196 and 36–92 ng/ml.

## Discussion

### Main Findings and Clinical Significance

In patients with incident atrial fibrillation, race/ethnicity was reported to be independently associated with initiating any anticoagulant therapy and direct-acting oral anticoagulant use among anticoagulant initiators. Moreover, limited data is available for testing of coagulation in patients treated with non-vitamin K antagonist oral anticoagulants in Chinese populations. We present a multi-center study of more than 1,000 blood samples from 301 subjects, assessing pharmacokinetics and PD of dabigatran. The more novel data is the expected peak and trough levels range for Asian patients taking dabigatran.

In the study, HTI had good correlation with HPLC-MS/MS at all collection time (*p* < 0.001), and we used Sysmex reagent to ensure the accuracy of detection results at low concentrations. In addition, the HTI method only takes 5 min, providing a reliable technical method for rapid and accurate clinical evaluation of dabigatran concentration. Thus, HIT assay can be a good quantitative detection method for dabigatran in Asian population. The expected peak and trough levels (ng/ml) range derived from HTI assay of dabigatran 110 mg were 95–196 and 36–92.

### Dabigatran Monitoring in Clinical Practice

Clinical physicians prescribed the 110 mg dose instead of the 150 mg dose in Asian patient group, several reasons were taken in consideration for this. Firstly, Asians are smaller in body size and have lower body mass index than non-Asians: in the subgroup analysis of the RE-LY trial, the Asians have substantially lower body weight than the non-Asians (66 vs. 86 kg) (24). Therefore, the clinical physicians in China/Asian tend to prescribe a lower dose of dabigatran. Secondly, Asians have a high risk for warfarin-related intracranial hemorrhage when compared with whites (*HR*, 4.06) (25), this make physicians more conservative in prescribing other new anticoagulants as well.

Quantitative measurements of dabigatran are gaining importance in emergency and special situations, especially in the low concentration range. APTT or PT are influenced by biological factors or method-specific components (26). The HTI and ECT assays are specific modified thrombin clotting time assays, thus they are less likely to be influenced by the above factors. Dabigatran concentration was reported to be determined using the thrombin-based Hemoclot coagulation assay, similar to our patients (27).

Apart from HPLC-MS/MS, studies measured dabigatran concentrations *via* HTI and ECA (28). They suggested that HTI showed good agreement with HPLC-MS, in line with results observed in our study. In addition, HTI had good sensitivity and specificity in determination of dabigatran at the 50 ng/ml, but with impaired sensitivity (73%) at the 30 ng/ml. In our study, dabigatran concentrations lower than the 30 ng/ml can also be determined by a low range HTI calibrator. In another study (16), the dilute thrombin time and chromogenic and ECA were proved to accurately identify therapeutic and supratherapeutic dabigatran levels. And, the study demonstrated that the reference detection range of dabigatran 150 mg was 27–411 ng/ml in American population. Inconsistent with their findings, in our study, the reference detection range of single dose dabigatran 150 mg in healthy subjects was 23–159 ng/ml; The expected peak and trough levels (ng/ml) range derived from HTI assay of dabigatran 110 mg were 95–196 and 36–92. The results of this study can provide a quantitative method for reference and the expected trough and peak concentrations for Asian patients taking dabigatran.

In the guidelines of 2019 “International Council for Standardization in Hematology Recommendations for Hemostasis Critical Values, Tests, and Reporting (ICSH),” the plasma concentration ranges of dabigatran are mainly from a few literatures. A multiple logistic regression model showed that ischemic events was inversely related to trough dabigatran concentrations (c-statistic 0.66, 95% CI 0.61–0.71, *p* = 0.045), with age and previous stroke (*p* < 0.0001) as significant covariates (29). Multiple logistic regression showed major bleeding risk increased with dabigatran exposure (c-statistic 0.72, 95% CI 0.69–0.74, *p* < 0.0001), while age, ASA use, and diabetes as significant covariates (*p* < 0.0001, <0.0003, 0.018). Moreover, limited information exists on their performance and expected peak/trough levels, especially in the ability to measure low/high concentrations of Asian population accurately. In this study, a multi-center quantitative assay of dabigatran was conducted for the first time in the Chinese population, providing a basis for the rational use of dabigatran in the Asian population, and also providing important Asian population data for the update of the international clinical guidelines for hematological testing.

The range for peak and trough levels of dabigatran is wide according to the guideline and our study. Some potential reasons and measures might affect the range and accuracy of the drug concentration. Previous study reported that bodyweight and serum creatinine were key factors in predicting trough concentration (30). Collectively, present study demonstrated that large variability in dabigatran concentration could be predicted by age, BMI and history of heart failure based on the data from 86 Chinese patients with NVAF receiving 110 mg dabigatran bid (31). Moreover, dabigatran monitoring was also Influenced by TT reagent with different thrombin concentrations: Sysmex-TT was very sensitive at low concentrations of dabigatran (0–100 ng/mL), while Instrument Laboratory (TT-5 ml) and Stago-TT were sensitive at medium concentrations of dabigatran (0–300 ng/ml), and Instrument Laboratory (TT-2 ml) was less sensitive for a wide concentration of dabigatran (0–500 ng/ml; *p* = 0.007) (32).

In our study, we used Sysmex reagent to ensure the accuracy of detection results at low concentrations, and the HTI assay only takes 5 min, providing a reliable technical method for rapid and accurate clinical evaluation of dabigatran concentration.

### Limitations

This study has some limitations. Reagents from different manufacturers might affect the results, such as Stago, Werfen, and Siemens. Second, we monitored trough and peak levels of dabigatran, but the interindividual coefficient of variability was not examined in the study.

## Conclusion

In the study, we present a multi-center study of more than 1,000 blood samples from 301 subjects on assessment pharmacokinetics of dabigatran, and PD with different assays. The more novel data are the expected peak and trough levels range for Asian patients taking dabigatran, and provided important Asian population data for the update of the international clinical guidelines for hematological testing.

## Data Availability Statement

The original contributions presented in this study are included in the article/supplementary material, further inquiries can be directed to the corresponding authors.

## Ethics Statement

The studies involving human participants were reviewed and approved by an independent Ethics Committee and the Institutional Review Board of Peking University First Hospital and all participating research sub-central hospitals. The patients/participants provided their written informed consent to participate in this study.

## Author Contributions

YC, QX, and JJ: conception and design. ZL, QfX, QX, HZ, and GM: provision of study materials or patients and collection and assembly of data. ZL, QfX, HZ, and QX: data analysis and interpretation. All authors: manuscript writing and Final approval of manuscript.

## Conflict of Interest

The authors declare that the research was conducted in the absence of any commercial or financial relationships that could be construed as a potential conflict of interest.

## Publisher’s Note

All claims expressed in this article are solely those of the authors and do not necessarily represent those of their affiliated organizations, or those of the publisher, the editors and the reviewers. Any product that may be evaluated in this article, or claim that may be made by its manufacturer, is not guaranteed or endorsed by the publisher.
